# The challenge and response of mental health institutions in COVID-19 pandemic: from chaos to new normal

**DOI:** 10.1038/s41398-020-01059-y

**Published:** 2020-11-06

**Authors:** Gang Wang, Lina Wang, Xuebing Liu, Yuping Ning, Wei Hao

**Affiliations:** 1 Wuhan Mental Health Center, The Ninth Clinical School, Tongji Medical College, Huazhong University of Science and Technology, Wuhan, China; 2 Tianjin Mental Health Center, Tianjin, China; 3 Affiliated Brain Hospital of Guangzhou Medical University (Guangzhou Huiai Hospital), the First School of Clinical Medicine, Southern Medical University, Psychiatry Department, Guangzhou, China; 4 Mental Health Institute of the Second Xiangya Hospital, Central South University, National Clinical Research Center on Mental Disorders, Changsha, China

**Keywords:** Schizophrenia, Scientific community

Coronavirus disease 2019 (COVID-19) shows a global outbreak^1,2^. Patients with mental disorders are categorized as a vulnerable group that faces a high risk of the disease. COVID-19 has a heavy impact on mental health^3^. Mental health institutions in China, the first country to report COVID-19, have encountered unprecedented difficulties and challenges. In this article, we provide a brief summary of the challenges and lessons learned by first-line mental health staff in mental health institutions in China during this period, and we propose suggestions to deal with similar challenges in the future.

## Chaos and challenge

The pandemic has disrupted the normal medical service model. In the early stage of the pandemic, many mental health institutions could not provide services normally. Patients had difficulty seeing doctors and obtaining psychotropic medicine, leading to a significant increase in mental symptom relapses, substance abuse, and related aggressive and violent behaviors. Many caregivers called the doctor, and it was increasingly difficult to manage patients due to relapse. Because acute psychotic disorders were not treated in a timely manner, some patients with medical issues could not be treated promptly. In the late stage, many psychiatric patients with serious psychotic symptoms and physical conditions flooded hospitals, which greatly increased the risk of COVID-19 exposure and the difficulty faced by medical staff in providing treatment.

In the early stage of the outbreak, similar to other medical institutions, mental health institutions were not adequately^4^ prepared due to the lack of effective measures to prevent nosocomial infection of COVID-19. The city at the epicenter of the outbreak, Wuhan, presented clustered infections in health professionals and patients. Due to the lack of comprehensive diagnosis and treatment capacity in psychiatric hospitals, psychiatric patients with infection or medical diseases could not be treated effectively or promptly.

In the early stage, the government and social organizations mainly focused on controlling the spread of the pandemic. Thus, there was insufficient attention to and investment in the mental diseases of the general population and those with high risk needing attention (such as medical staff in pandemic areas), and mental health services could not be provided in a timely manner.

## Response effort and new normal

In the face of these challenges, mental health agencies, in coordination with the government, have actively undertaken the following work.

1. Building transitional psychiatric wards: Based on the criteria for building isolation wards for infectious diseases, transitional wards were established to screen and determine whether newly admitted inpatients were infected with COVID-19. Inpatients were assigned to infectious isolation wards or general wards based on the screening results.
2. Building isolation wards for patients with COVID-19 infection: after the pandemic was declared, the Wuhan government made an overall arrangement to reconstruct the isolation wards of designated large psychiatric hospitals through financial aid, such that psychiatric patients could be treated promptly.
3. Newly diagnosed COVID-19 patients with psychosis in Wuhan were transferred to psychiatric hospitals with isolation wards to treat mental illness after their pneumonia prognosis had improved, and such patients were discharged once their mental and physical symptoms were alleviated. We also built a psychiatric ward in an infectious disease hospital for the treatment of mental illness patients with serious COVID-19 complications.
4. Alleviating the difficulty of seeing doctors: The National Health Commission has temporarily adjusted the prescription regulations of outpatients, allowing us to extend the prescription amount of disposable psychotropic medicines and narcotic drugs and allowing patients to have drugs shipped to them after visiting authorized medical websites. Additionally, a community was appointed to be responsible for drug distribution and monitoring the relapse of methadone patients under its jurisdiction.
5. Strengthening the functioning of academic communities: China has issued several guidelines for COVID-19-related psychiatric crisis intervention and expert consensus to guide clinical work^5^. Crisis intervention teams and counseling hotline services were set up to address psychological problems caused by the pandemic^6^.

The above measures effectively contained the spread of COVID-19 in psychiatric hospitals, and there were no reports of clustered infections in the middle and late stages of the pandemic. Early and timely mental health counseling and hotline services, as well as timely management of patients with acute psychotic symptoms in the later stage, significantly reduced the aggressive and violent behaviors and deaths of patients due to mental problems.

## Recommendations

The COVID-19 pandemic poses a major challenge for the service provision of mental health institutions. Based on our experience, we make the following recommendations on how to deal with the challenges that may arise in the future:

1. Strengthening the concept of integrated multidisciplinary medical service: the pandemic exposed the limitations of mental health institutions in dealing with infectious and medical diseases. According to statistical data, 70% of the patients in isolation and observation wards in the Wuhan Mental Health Center, Tianjin Anding Hospital, and other regions were accompanied by a variety of medical diseases; however, the psychiatric staff did not have sufficient updated knowledge about dealing with such issues. Thus, we suggest that a multidisciplinary medical team should be organized when formulating emergency medical disposal plans in the future to enhance the ability to deal with physical diseases.
2. Strengthening community-based mental health services: Although communities play a prominent role in combating a pandemic, the management of mental illness is limited. If communities and mental health institutions effectively cooperate to strengthen their role in prevention, the bottleneck problems encountered in early detection, diagnosis, treatment, and referral will be resolved. Therefore, it is imperative to establish a seamless connection between communities and mental health facilities and to improve community-based medical services for mental health.
3. Making full use of information technology (IT): the Internet and mobile devices have great advantages in mental health services and remote consultation, alleviating the difficulties that patients face in medical treatment and the difficulties in isolation ward management^7^.
4. Strengthening the coordinating role of the government: government sectors should be involved in emergency response, professional training, referral, and other programs. Simultaneously, international experience exchange should be strengthened, and experiences and lessons in scientific research and in clinical and public health encountered in psychiatry should be shared in a timely manner through academic reports and conferences. Thus, all efforts could be combined to efficiently fight the pandemic under the crisis brought by limited medical resources.

We presume that after the pandemic, the service model of the mental health system will change, and new medical methods might pose some challenges to the traditional medical model. Thus, we need to prepare in advance. However, such changes will not be completed in a short duration and should be constantly explored to adapt to the requirements of the new era.
